# Intron retention-induced neoantigen load correlates with unfavorable prognosis in multiple myeloma

**DOI:** 10.1038/s41388-021-02005-y

**Published:** 2021-09-09

**Authors:** Chuanpeng Dong, Annamaria Cesarano, Giuseppe Bombaci, Jill L. Reiter, Christina Y. Yu, Yue Wang, Zhaoyang Jiang, Mohammad Abu Zaid, Kun Huang, Xiongbin Lu, Brian A. Walker, Fabiana Perna, Yunlong Liu

**Affiliations:** 1 Center for Computational Biology and Bioinformatics, Indiana University School of Medicine, Indianapolis, IN 46202 USA; 2 Department of BioHealth Informatics, School of Informatics and Computing, Indiana University-Purdue University Indianapolis, Indianapolis, IN 46202 USA; 3 Department of Medicine, School of Medicine, Indiana University, Indianapolis, IN 46202 USA; 4 Department of Medical and Molecular Genetics, Indiana University School of Medicine, Indianapolis, IN 46202 USA; 5 Zionsville Community High School, Boone, IN 46077 USA; 6 Melvin and Bren Simon Cancer Center, Indiana University School of Medicine, Indianapolis, IN 46202 USA; 7 Department of Biostatistics and Health Data Science, Indiana University School of Medicine, Indianapolis, IN 46202 USA

**Keywords:** Myeloma, Tumour biomarkers

## Abstract

Neoantigen peptides arising from genetic alterations may serve as targets for personalized cancer vaccines and as positive predictors of response to immune checkpoint therapy. Mutations in genes regulating RNA splicing are common in hematological malignancies leading to dysregulated splicing and intron retention (IR). In this study, we investigated IR as a potential source of tumor neoantigens in multiple myeloma (MM) patients and the relationship of IR-induced neoantigens (IR-neoAg) with clinical outcomes. MM-specific IR events were identified in RNA-sequencing data from the Multiple Myeloma Research Foundation CoMMpass study after removing IR events that also occurred in normal plasma cells. We quantified the IR-neoAg load by assessing IR-induced novel peptides that were predicted to bind to major histocompatibility complex (MHC) molecules. We found that high IR-neoAg load was associated with poor overall survival in both newly diagnosed and relapsed MM patients. Further analyses revealed that poor outcome in MM patients with high IR-neoAg load was associated with high expression levels of T-cell co-inhibitory molecules and elevated interferon signaling activity. We also found that MM cells exhibiting high IR levels had lower MHC-II protein abundance and treatment of MM cells with a spliceosome inhibitor resulted in increased MHC-I protein abundance. Our findings suggest that IR-neoAg may represent a novel biomarker of MM patient clinical outcome and further that targeting RNA splicing may serve as a potential therapeutic strategy to prevent MM immune escape and promote response to checkpoint blockade.

## Introduction

Multiple Myeloma (MM) is characterized by the clonal expansion of malignant plasma cells in the bone marrow [1]. Recent therapeutic advances have extended overall survival, but most MM patients ultimately relapse [2]. Immune checkpoint blockade (ICB) therapy has revolutionized the treatment of many solid tumors by harnessing the immune system for effective anticancer treatment [3]. In these diseases, clinical response to ICB therapy is associated with the presence of tumor-specific antigenic peptides, or neoantigens [4], a source of potential neoepitopes that can be loaded onto major histocompatibility complex (MHC) class I molecules to generate an antitumor immune response [5]. Cytotoxic T-cells recognize tumor neoantigens as foreign and kill the presenting tumor cells, which initiates an antitumor immunological memory that hinders tumor recurrence. An important source of cancer neoantigens is somatic DNA mutations in the genome’s coding regions [6] and the mutation-derived neoantigen load in several types of solid tumors corresponds with a better prognosis [7–10]. However, MM has a relatively low mutation frequency. In contrast to solid tumors, mutation-derived neoantigen load in MM has been associated with unfavorable outcome [11, 12].

Another potential source of tumor neoantigens is aberrant RNA splicing [13–16]. Alternative splicing is a regulatory mechanism that generates multiple mRNA transcripts from a single gene and significantly expands proteome diversity [17]. Consequently, disruption of splicing mechanisms has a large impact on the transcriptome and is a significant driver of disease [18]. Intron retention (IR) occurs when the spliceosome fails to remove specific introns from pre-mRNA molecules, and they remain in the mature polyadenylated mRNA. In normal cells, IR functions to further reduce the levels of relatively low abundance transcripts that are not needed in specific cell types, such as the expression of developmentally regulated genes [19, 20]. This type of regulation has been termed transcriptome-tuning and is brought about through both nuclear RNA degradation and nonsense-mediated mRNA decay [21].

IR occurs more frequently in nearly all cancer types compared with normal control tissues, even in the absence of DNA mutations in genes encoding proteins involved in splicing. Additionally, in cancer cells, transcripts with IR are present at relatively high levels in cytoplasmic mRNA [22]. These transcripts are translated and degraded by the nonsense-mediated decay (NMD) pathway, a translation-coupled mechanism that eliminates mRNAs containing premature translation-termination codons [23]. Although most IR transcripts are subject to NMD-induced degradation, this process does not occur until after the pioneer round of translation, which can result in the production of neopeptides that bind to MHC molecules [24, 25]. Therefore, we hypothesized that IR-neoAgs in MM might impact immune response.

Herein, we used RNA-seq data from the MM Research Foundation (MMRF) CoMMpass study to identify IR events and predict IR-neoAgs. We found cells in bone marrow aspirates from MM patients exhibited high levels of IR events. However, consistent with the findings that high mutation-neoantigen load predicts unfavorable prognosis, high IR-neoAg load was correlated with shorter overall survival (OS) in MM. To investigate why high IR-neoAg load was not correlated with better MM patient survival, we performed gene set enrichment analysis on MM samples with high versus low IR-neoAg load. This analysis revealed that high IR-neoAg load was positively associated with the expression of T-cell inhibitory molecules, such as those involved in interferon (IFN) and tumor necrosis factor (TNF) alpha signaling activity. In addition, flow-cytometric analyses of four MM cell lines showed an inverse correlation between IR levels and MHC-II abundance, while treatment with a splicing inhibitor increased MHC-I protein abundance, especially in MM cells bearing high IR levels.

## Results

### Genes involved in spliceosome activities are differentially expressed between MM and normal plasma cells

**Fig. 1:**
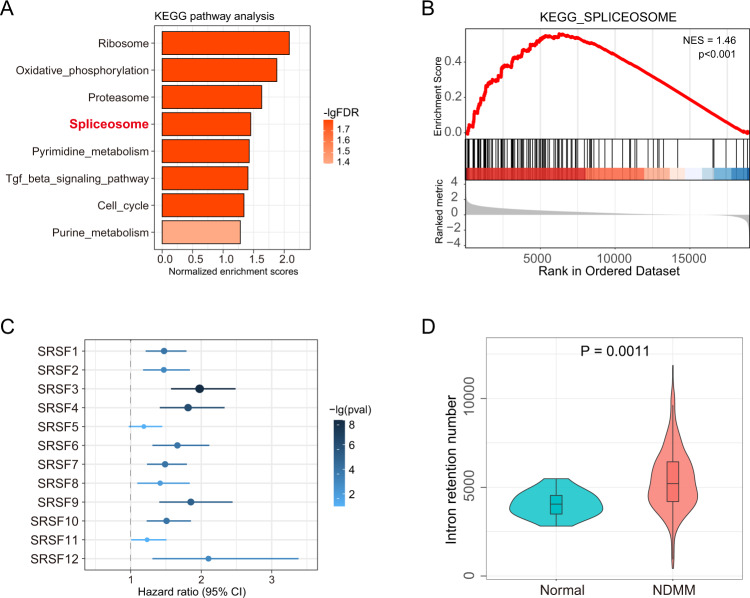
Intron-retention (IR) events in plasma cells from multiple myeloma (MM) patients are associated with altered RNA splicing. **A** Spliceosome is among the top significant pathways involving upregulated genes in newly diagnosed MM compared with healthy controls from GSE110486. **B** Gene enrichment plot for spliceosome pathway genes in MM samples compared with healthy controls from GSE110486. NES, normalized enrichment score. **C** Serine and arginine-rich splicing factor (SRSF) gene expression was associated with shorter overall survival time in MM; results were obtained from 767 newly diagnosed multiple myeloma (NDMM) patients in the MMRF cohort. **D** Comparison of the number of IR events in primary MM samples from MMRF (n = 767) compared with normal plasma cell samples from GEO (*n* = 13). Violin plots show the median and 25th and 75th percentiles (box) and the 95% confidence interval (whiskers). *P* value was determined using the Mann-Whitney test.

To investigate whether the expression of genes involved in RNA splicing was altered in MM compared to normal plasma cells, we analyzed differentially expressed genes using RNA-seq data of plasma cells from five newly diagnosed MM patients (NDMM) and five healthy controls (GSE110486). These results showed that the spliceosome pathway was among the top upregulated pathways in MM (Fig. 1A), where 67 out of 126 genes in the spliceosome pathway were upregulated significantly in MM samples. Gene set enrichment analysis also demonstrated that the spliceosome pathway was enriched in MM samples with a normalized enrichment score of 1.46 (*p* value < 0.001, FDR = 0.016, Fig. 1B). We also found that the 230 upregulated differentially expressed genes identified in GSE110486 were also highly enriched in the NDMM samples from the MMRF cohort, as compared to the normal plasma cells (Fig. S1A). In addition, the expression levels of 11 out of 12 serine and arginine-rich splicing factor (SRSF) protein genes, a conserved family of proteins involved in RNA splicing, were upregulated in MM (Fig. S1B). Additional analysis of the MMRF data suggested that the increased expression of each of these 12 SRSF family genes was associated with decreased overall survival time (Fig. 1C).

### IR events are more common in MM compared to control plasma cells

Accumulating studies provide strong evidence that IR is an important source of tumor neoantigens [15]. We sought to characterize IR in MM and its association with MM progression. The number of IR events and their expression levels were assessed for both the MM and control samples in GSE110486. We observed an average of 5799 IR events in five MM samples and 4761 IR events in healthy controls (Fig. S1C); however, due to the limited sample size, this difference did not reach statistical significance. Next, we compared the number of IR events in 767 NDMM samples from the MMRF cohort with 13 control plasma cell samples from bone marrow and tonsil of healthy subjects (five from GSE110486 and eight from GSE114816). The NDMM samples showed more IR events with an average of 5391 per sample compared to 4065 IR events in the normal plasma cells. The result of this comparison was statistically significant (Wilcoxon test, *p* value = 0.001) (Fig. 1D).

### IR-neoAgs are abundant in multiple myeloma

**Fig. 2:**
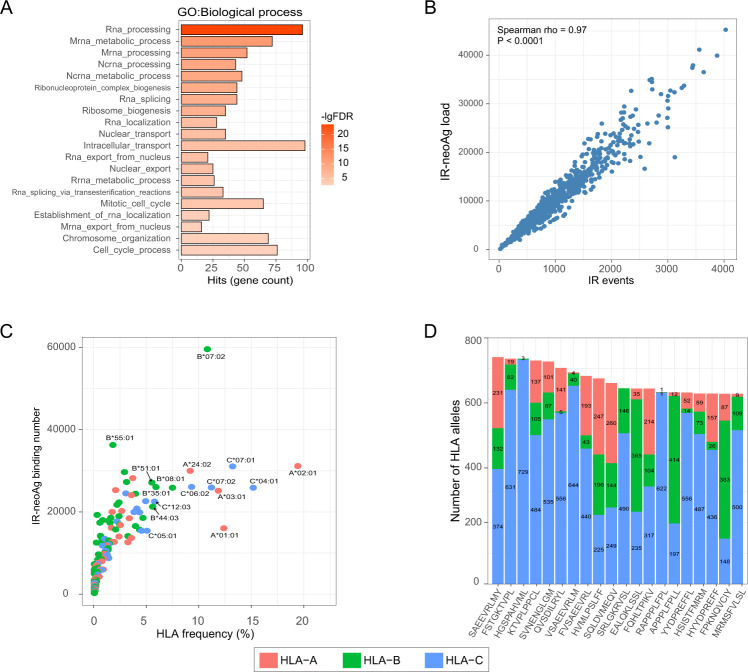
MM-specific IR-neoAgs. **A** Gene ontology enrichment analysis of genes harboring MM-specific IR events in specialized biological processes. **B** Scatter plot showing the number of IR events vs. IR-neoAg load in NDMM patients in the MMRF cohort. Each dot represents an individual patient (*N* = 767). Spearman correlation rho = 0.97, *p* value < 0.0001. **C** Scatterplot showing *HLA* allele frequency vs. the number of predicted IR-neoAgs bound in MMRF NDMM samples. **D** Top 20 most abundant IR-neoAg peptides and the number of *HLA* alleles in the MMRF NDMM samples predicted to bind each peptide; numbers in each bar represent the quantity of *HLA* allele subtypes.

Emerging evidence suggested that IR events in the cancer genome can be a source for immunogenic peptides [24]. Therefore, we investigated the potential for IR events to produce neoantigens in MM. To begin to address this question, we filtered the IR events that also occurred in normal plasma cells from the events identified in MMRF RNA-seq data. IR events occurring in normal plasma cells were removed because they were not expected to produce immunogenic peptides due to host immune tolerance. To identify the IR events in the healthy plasma cells, we analyzed RNA-seq data from the 13 plasma cell samples in GSE110486 and GSE114816. We detected a total of 9715 IR events that appeared in at least one healthy control sample; these IR events were eliminated from the list of events identified in the MM samples (Table S1). After filtering the normal IR events, the average number of MM-specific IR events per sample was 1009 and ranged from 21 to 4138 (Table S1). Interestingly, gene ontology analysis of 450 genes harboring MM-specific IR events that occurred in more than half of the NDMM samples showed that these genes were enriched in pathways involving RNA processing and RNA transport (Fig. 2A).

To computationally predict IR-neoAgs, we first determined the *HLA-I* genotype of each MMRF patient using the RNA-seq data. A total of 178 unique *HLA-A/B/C* alleles were identified from 767 individual patients of the MMRF cohort (*HLA* alleles and their frequencies are listed in Supplementary Table S2). Next, the retained intron sequences were translated into protein sequences, which were then segmented into 8–11 amino acid peptides, where at least one amino acid was translated from the intronic region. Any peptide that could also be generated from normal proteins was further removed. The remaining IR-derived neopeptides were then evaluated for their predicted binding affinity with the set of patient-specific *HLA* alleles using NetMHCpan (v4.1). Peptides with a NetMHCpan predicted rank score less than two (the default cutoff from NetMHCpan) were selected as IR-neoAgs. IR-neoAgs were called for 893 RNA-seq samples from the MMRF cohort (including both newly diagnosed and relapsed samples). Not surprisingly, the number of IR events and the IR-neoAg load were highly correlated (Spearman correlation rho = 0.97, *p* value < 0.0001, Fig. 2B). We further evaluated whether any *HLA* allele presented more IR-neoAgs than other alleles at the population level (Fig. 2C). Our results revealed that *HLA-B07:02* presented the highest number of neoantigen peptides (*N* = 59 588); this allele was detected in 10.8% of samples. The most common allele, *HLA-A02:01* which was detected in 19.4% of the samples, presenting 31,161 IR-derived peptides.

Notably, 24,680 out of 479,685 of the IR-neoAgs were shared across more than 5% of the multiple MM samples. This observation would suggest that there might be potential for developing cancer vaccines in the future based on IR-neoAgs. We also found that 20 neoantigens occurring in more than 80% of NDMM samples were preferentially presented by *HLA-C* alleles (Fig. 2D), suggesting neoantigens presented by HLA-C alleles could be prioritized for cancer vaccine development.

### IR-neoAg load correlates with unfavorable clinical outcome

**Fig. 3:**
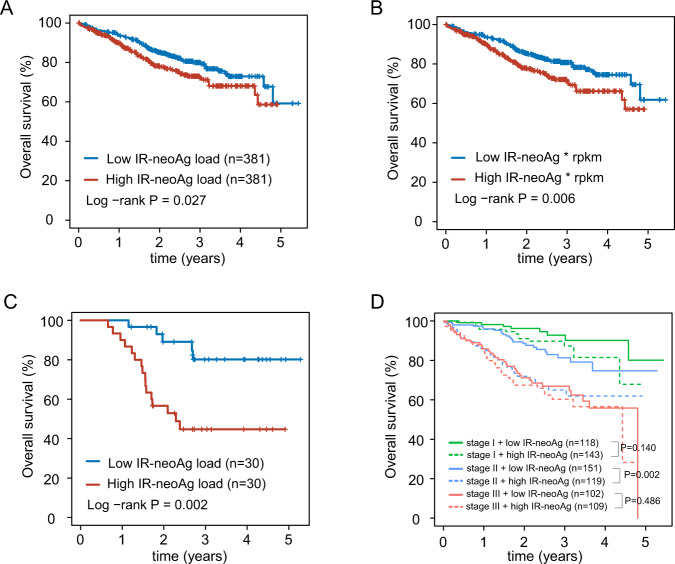
Association of IR-neoAg load with overall survival in the MMRF cohort. Kaplan–Meier survival curves comparing: **A** NDMM patients with high (defined as above the median) IR-neoAg load to those with low (below the median) IR-neoAg load; **B** NDMM patients with high or low expression levels of IR-neoAgs that were quantified using RPKM values of the source IR events; **C** MMRF patients with relapsed disease and either high or low IR-neoAg load; and **D** high and low IR-neoAg load subdivided by ISS disease stage.

We next asked whether IR-neoAg load was associated with overall survival (OS) in the MMRF cohort. Kaplan-Meier survival analysis revealed that NDMM patients with higher than the median IR-neoAg load had significantly shorter OS (log-rank test, *P* = 0.027, Fig. 3A). When considering the expression levels of IR-neoAgs, we observed an even more substantial prognostic effect, with a *p* value reaching 0.006 (Fig. 3B). Similarly, higher than the median IR-neoAg load predicted shorter OS for MM patients at the time of first relapse (log-rank test, *P* = 0.002, Fig. 3C, *n* = 60). Notably, relapsed MM samples with lower IR-neoAg load had a higher 2-year OS rate compared to patients with higher IR-neoAg load (OS 0.85 vs. 0.57).

To determine whether IR-neoAg load was associated with clinical features of MM, we asked whether IR-neoAg load correlated with the International Staging System (ISS) [26], which is a reproducible predictor of MM outcome. We did not find that IR-neoAg load was associated with the ISS disease stage in NDMM from the MMRF cohort (one-way ANOVA *P* = 0.724, Fig. S2A). To determine whether the addition of IR-neoAg load to the ISS stage improved the prediction of OS, we performed survival analysis on patients stratified by disease stage and IR-neoAg load. This analysis showed that stage II MM patients with higher than the median IR-neoAg load had significantly shorter OS than stage II patients with low IR-neoAg load (log-rank test, *P* = 0.002, Fig. 3D). A similar trend was observed with stage I patients, although the association did not reach statistical significance (*P* = 0.14). IR-neoAg load had no apparent prognostic value for OS in stage III MM patients (*P* = 0.486). In addition to ISS stages, chromosomal hyperdiploidy (HRD) is widely used in defining genetic subtypes of MM patients, and HRD-myeloma is associated with better survival compared to nonhyperdiploid (nHRD) MM [27]. Although we observed that higher than the median IR-neoAg load was apparently associated with shorter OS in both HRD and nHRD MM patients, these associations did not reach statistical significance (Fig. S2B).

We further examined whether the prognostic performance of IR-neoAg load was independent of other clinical factors. We used multivariate Cox analysis to test the performance of IR-neoAg load after adjusting for other clinical factors, including age, sex, P53 status, ISS stage, as well as the revised ISS stage after adjusting for lactate dehydrogenase (LDH) level, chromosomal aberrations, and other factors. In the multivariate analysis, the hazard ratio of high versus low IR-neoAg load for OS in NDMM was 1.491 (*p* value = 0.027; 95% CI 1.056 to 2.492) (Table 1), indicating that the IR-neoAg load offers prognostic power that is independent of other clinical factors.

**Table 1:** Univariate and multivariate Cox regression analysis of OS in newly diagnosed MM.

	Univariate analysis			Multivariate analysis		
	HR	95% CI of HR	*P* value	HR	95% CI of HR	*P* value
**Variable**						
IR-neoantigen (high/low)	1.431	1.041–1.968	0.027	1.622	1.056–2.492	**0.027**
Age (years)	1.038	1.021–1.055	<0.001	1.046	1.023–1.069	**<0.001**
Sex (male/female)	1.536	1.089–2.165	0.014	1.537	0.954–2.476	0.077
**Stage**						
ISS Stage (I/II/III)	2.038	1.640–2.532	<0.001	1.442	0.963–2.159	0.076
Revised ISS Stage	2.398	1.760–3.266	<0.001	1.496	0.865–2.588	0.15
**TP53 status**						
TP53_Loss	1.088	0.823–1.438	0.555	1.343	0.806–2.238	0.257
BI_TP53	0.622	0.450–0.859	0.004	0.652	0.206–2.062	0.467
NS_TP53	2.755	1.603– 4.732	<0.001	1.153	0.145–9.146	0.893

IR-neoantigen, Intron retention-derived neoantigen, ISS stage Myeloma International Staging System, HR Hazard ratio, CI confidence interval, Revised Stage (R-ISS) was calculated as defined by the International Myeloma Working Group, by considering LDH, β2-microglobulin, albumin, deletion of chromosome 17p, and translocations; TP53_Loss: TP53 copy number variation; BI_TP53: bi-allelic p53 status; NS_TP53: presence of nonsynonymous mutation on TP53.

Bold values indicate *p* values < 0.05. Multivariate analysis *p*-value for age is 0.00006.

### Higher T cell inhibitory signals associate with IR-neoAg and poor prognosis in MM

Our observation that higher IR-neoAg load was associated with shorter OS is consistent with previous reports of mutation-derived neoantigen load in MM [11, 12]. However, this finding is the reverse of previously reported observations that high mutation-derived and IR-neoAg loads are associated with longer OS in patients with solid tumors, including melanoma [7], lung cancer [8], breast cancer [10], and pancreatic cancer [6]. In addition, there is increasing evidence that T cells present in the MM microenvironment show an exhausted and suppressed phenotype [28]. This would suggest that additional changes in MM plasma cells may affect the anti-MM immune response.

**Fig. 4:**
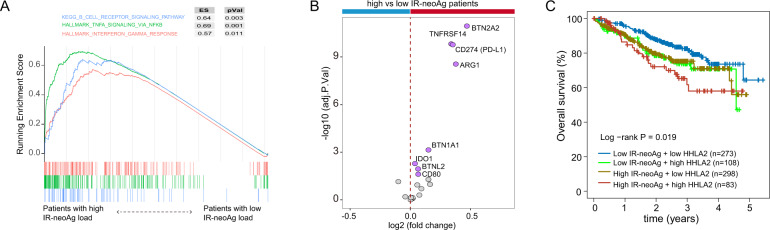
High T-cell inhibitory signature in MMRF patient cohort. **A** Gene set enrichment analysis comparing NDMM patients with high and low IR-neoAg loads. Pathways involved in T-cell suppression and B-cell receptor signaling were enriched in patients with high IR-neoAg load. **B** T-cell signaling co-inhibitory genes were upregulated in patients with high IR-neoAg load. The genes with adjusted *p* value < 0.05 are labeled in purple; the blue bar indicates downregulated genes in NDMM patient samples with high IR-neoAg load and the red bar indicates upregulated genes. **C** Kaplan–Meier survival curves showing overall survival in MMRF cohort patients with high (>= 75%) and low (< 75%) expression of *HHLA2* and high (above the median) or low (below the median) IR-neoAg load.

To test this hypothesis, we conducted differential expression and gene set enrichment analysis on RNA-seq data from the MMRF cohort, comparing samples from NDMM patients with either higher or lower than the median IR-neoAg load. Notably, we observed a significant enrichment of the pathways related to T-cell suppression. We found that IFN gamma signaling and TNFα signaling via NF-κB pathways were upregulated in patients with high IR-neoAg load (Fig. 4A). These pathways are involved in the recruitment of T-regulatory (Treg) cells that control cytotoxic T-cell killing. In addition, the B-cell receptor (BCR) signaling pathway was significantly enriched in patients with high IR-neoAg loads. Previous studies demonstrated that sustained activation of BCR signaling plays critical roles in B-cell malignancies [29]. This result suggests that molecular features in cells with higher IR-neoAg load might contribute to T-cell and B-cell dysfunction in MM.

Based on this finding, we postulated that increased expression of T-cell co-inhibitory molecules in MM cells exhibiting high IR-neoAg load might be a partial explanation for the reduced antitumor immunity and thereby facilitate cancer immune evasion [30]. These co-inhibitory molecules function as brakes to inhibit T-cell activation. Higher expression levels of co-inhibitory ligands on the cancer cell surface can negatively impact T-cell function. To begin to address this question, we first analyzed the expression levels of 20 co-inhibitory genes identified by Dufva and colleagues [31], which include genes for eight B7 ligands, six enzymes impacting T-cell activity, and six other genes from the butyrophilins and CD226 family (Table S3). We found that these co-inhibitory genes tend to have higher expression levels in NDMM samples with higher IR-neoAg load (Fig. 4B). We found that *CD274* (PD-L1) expression was 1.3-fold higher in patients with high IR-neoAg load (adjusted *p* value < 0.0001), suggesting there could be a stronger immune suppression in patients with higher IR-neoAg load. Next, we analyzed co-inhibitory gene expression from 29 MM cell lines compared to other cancer cell lines in the Cancer Cell Line Encyclopedia (CCLE). Surprisingly, we found that the average expression level of PD-L1 in the MM cell lines was lower than most other types of cancer cell lines (Fig. S3), which might partially explain why anti-PD1 therapy has had a limited response rate in MM. Other B7 co-inhibitory ligands, such as *CD86*, *CD80*, and *HHLA2*, showed high expression levels in myeloma cell lines relative to the other cancer cell lines, implying that these B7 ligands might serve as potential targets for immune checkpoint therapy. Kaplan–Meier survival analysis revealed that the patients with higher *HHLA2* and IR-neoAg load had the worst outcome (Fig. 4C), which provides further support that HHLA2 may be a druggable target for treating MM in the future [32].

### RNA splicing inhibition impacts MHC-I protein expression in MM cells

**Fig. 5:**
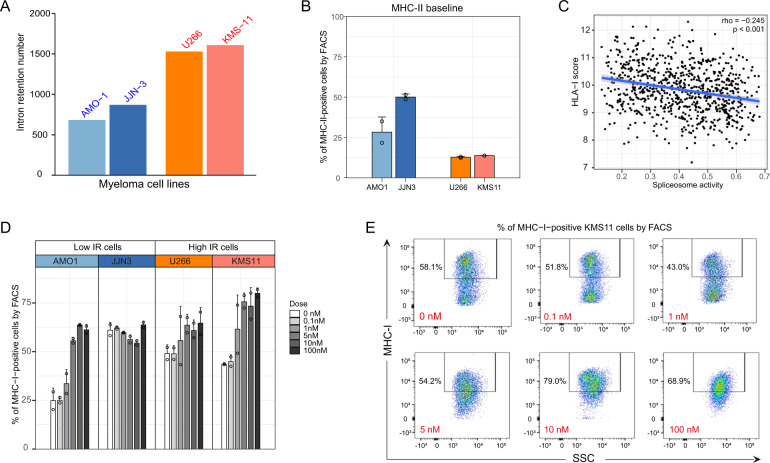
Intron retention, spliceosome activity, and MHC abundance in MM cells. **A** Number of intron retention events determined from CCLE RNA-seq data in a panel of four MM cell lines. **B** MM cell lines with high IR levels had lower baseline MHC-II cell surface expression determined by flow cytometry. **C** *HLA class I* gene score (average expression of *HLA-A/B/C* alleles) from MMRF RNA-seq data was negatively associated with spliceosome pathway activity. **D** MHC-I genes were upregulated in a dose-dependent manner in three of four MM cell lines following treatment with the splicing inhibitor pladienolide-B (0–100 nM) for 96 h. **E** Representative flow cytometry plots illustrating the percentage of KMS11 cells with increased MHC-I (*HLA-A/B/C*) gene expression following treatment with pladienolide-B (0–100 nM) for 96 h. Gates were set based on isotype controls and unstained controls.

MHC molecules encoded by the *HLA-I* and *HLA-II* genes are essential components in IR-neoAg presentation on the cell surface. Therefore, we investigated the relationship between IR events (IR levels) and MHC protein abundance in four MM cell lines, namely JJN3, U266, KMS11, and AMO1 cells. These cell lines were selected because KMS11 and U266 had the highest levels of IR, while JJN3 and AMO1 had the lowest levels of IR based on RNA-seq data from the CCLE consortium (Fig. 5A). We measured MHC-I and MHC-II cell surface abundance in these MM cells by flow cytometry before and after treatment with the splicing inhibitor pladienolide-B for 96 h.

As demonstrated in Fig. 5B, the basal cell surface level of MHC-II was lower in the KMS11 and U266 cell lines bearing higher IR levels, compared to the JJN3 and AMO1 cell lines bearing lower IR levels. Low MHC-II abundance in MM cells with high IR levels is consistent with our observation in MM patients where higher IR levels and low *HLA-II* gene mRNA expression was associated with worse clinical outcomes (data not shown).

Next, we investigated whether splicing activities, as measured by the mRNA expression levels of genes encoding key splicing factors and regulators, correlated with the mRNA expression levels of the *HLA* genes encoding MHC-I and MHC-II complexes in the MMRF RNA-seq data. We observed a negative correlation between the expression levels of MHC-I genes and spliceosome pathway activities, as measured by ssGSEA enrichment scores (Fig. 5C, Spearman correlation rho = −0.245, *p* < 0.001). No correlation was observed between the expression of genes encoding MHC-II molecules and spliceosome pathway activities (Fig. S4A).

To investigate whether low MHC-I expression might be a result of increased splicing activity, we treated MM cell lines with the splicing inhibitor pladienolide-B, which targets SF3B1, a gene encoding subunit 1 of the splicing factor 3b protein complex, and measured MHC-I cell surface expression by flow cytometry. We found that MHC-I expression levels were significantly increased in three of the 4 MM cell lines, including both cell lines with higher IR levels (Fig. 5D). As shown in Fig. 5E, MHC-I cell surface abundance in KMS11 MM cells exhibiting high IR increased following pladienolide-B treatment. This finding strongly suggests that modulation of splicing activity may regulate the abundance of MHC-I class proteins along with the antigen presentation potential in MM cells. Consistent with the lack of correlation between *HLA-II* gene expression and spliceosome pathway activity (Fig. S4A), no significant changes in MHC-II protein abundance were observed in the MM cell lines after splicing inhibition (Fig. S4B).

## Discussion

In this study, we demonstrate that intron retention is an important source of neoantigens in multiple myeloma, which impacts patient clinical outcome. We showed that newly diagnosed MM samples exhibited more intron retention events than normal plasma cells and that higher IR-neoAg load was significantly associated with unfavorable survival in both newly diagnosed and relapsed MM. Our findings indicate that bioinformatic predictions of immune recognition of neoantigens arising from genomic or transcriptomic alterations in MM might not be useful in selecting patients for immune checkpoint therapy. Further, our analyses revealed that poor outcome in MM patients with high IR-neoAg load is associated with higher expression levels of checkpoint genes and elevated IFN signaling activity, which implies strong T-cell suppression. Therefore, our results suggest a potential mechanism for MM cell immune evasion despite having an increased neoantigen load compared to normal plasma cells.

Whereas high neoantigen load generally predicts favorable survival and higher likelihood of response to checkpoint blockade in many solid tumors such as breast cancer [10], lung cancer [8], glioblastomas [9] and melanoma [7], we found that a high neoantigen load in MM patients was associated with poor prognosis [11]. In addition, the immune context of the bone marrow microenvironment is more complex compared with solid tumors, where cytokines and immune cell components in the bone marrow provide a unique seedbed for myeloma cell growth [33]. Therefore, the underlying mechanisms that allow for MM cell immune escape are apparently different from other tumors.

In addition to somatic DNA mutations, RNA alternative splicing, including intron retention, has been reported to be a novel source of neoantigens [24]. Numerous studies have reported that the splicing machinery is dysregulated in multiple cancer types, including bladder cancer [34], breast cancer [35], melanoma [36], prostate cancer [37] and hematological cancers [38, 39]. In addition, intron retention events have been observed frequently in prostate cancer [40] and pancreatic cancer [41]. Yang et al. reported that blood cells have a high level of splicing diversity compared to other tissues, next to testis, brain, and muscle-skeletal tissue, in the GTEx transcriptional data [42]. IR events represent a large proportion of alternative splicing events in blood tissue. These findings prompted us to investigate whether IR-neoAg could contribute to immune responses in MM, in particular to antigen presentation and T-cell mediate responses. We found that higher IR-neoAg load was significantly associated with shorter survival time, both in newly diagnosed and relapsed MM. This finding was further strengthened when the expression levels of IR-neoAg were considered (p-value reached 0.006).

Over the past decade, immune checkpoint blockade (ICB) therapy has revolutionized cancer therapy in several tumor types [43]. However, response to the immune checkpoint inhibitor pembrolizumab (anti-PD1) has been limited in MM [44]. Clinical response to ICB has been closely linked with the abundance of tumor-specific neoantigens, the presence of cytotoxic T-cell infiltration, and distinct tumor microenvironment profiles. Previous reports have demonstrated an increase in the mutation-derived neoantigen load in MM and have also confirmed a neoantigen T-cell response in relapsed patients with MM [12]. These results implied that a T-cell mediated immune response might be suppressed or impaired in MM. Zelle-Rieser et al. reported that CD8 + T-cells expressed several molecules associated with T-cell exhaustion (PD-1, CTLA-4, CD160) as well as the T-cell senescence marker CD57 at the MM tumor site [28, 45]. Our results showed that higher IR-neoAg load was positively correlated with higher expression levels of T-cell inhibitory molecules and genes belonging to the Tregs activating pathway. Dufva et al. reported that decreased *HLA*-*II* gene expression might be a potential immune evasion mechanism in hematological cancers [31]. We also found that gene expression levels of *HLA*-*II* genes were significantly lower in newly diagnosed MM compared with healthy control cells.

Despite these RNA-seq-based observations, direct evidence of IR-neoAg presentation on MM cells using immune-peptidomics technology could strengthen our conclusion. We hypothesize that standard MM treatment options do not generate an effective immune response that leverages the neoantigen immunotherapeutic potential. Indeed, we observed lower levels of MHC-II activity in MM cell lines with higher intron retention. In addition, we observed that MHC-I activity appeared to be inhibited in cells with elevated expression levels of splicing factors, a hallmark of MM, and that inhibition of spliceosome activity resulted in increased MHC-I activity. Collectively, these two mechanisms may partially explain why higher IR-induced neoantigen load in MM samples was not associated with better prognostic outcome. This result also suggests that splicing inhibitors could possibly boost the efficacy of immune checkpoint blockade therapy in MM by activating MHC-I presentation [46, 47]. Further analysis with integrated multi-omics data from different aspects of the immune landscape is needed to further understand the potential determinants of responsiveness to cancer immunotherapies in MM.

In conclusion, while neoantigen load has been associated with favorable survival in many solid cancers, our study strongly suggests that IR-neoAg load may serve as a clinically relevant risk factor that negatively impacts myeloma patient survival. Our analysis provides evidence that MM cells bearing high levels of IR-neoAgs also present T-cell inhibitory gene signatures, which may offset the neoantigen load in eliciting a cytotoxic T cell response. Moreover, we found that aberrant RNA splicing may also regulate MHC abundance and thus, contribute to MM immune escape. Our findings highlight the need to integrate multi-omics data to uncover the immune context and understand the factors that determine responsiveness of MM to immunotherapies. Also, this works suggests that targeting splicing may represent an additional therapeutic strategy to promote anti-MM immune response.

## Materials and methods

### RNA-seq data sets

The raw data from the MMRF study was obtained through an authorized data access request for dbGaP study accession: phs000748.v7. p4. RNA-seq data from 893 samples, including both newly diagnosed and relapsed subjects, were downloaded and converted to fastq format using SRA-tools (v2.10.0). Curated survival and clinical data were downloaded from the UCSC Xena cancer browser (http://xena.ucsc.edu) [48]. The revised International Staging System (R-ISS) was calculated as defined by the International Myeloma Working Group [49], by considering the presence of del(17p), t(4;14), and t(14;16) and information on serum β2-microglobulin, albumin, and lactate dehydrogenase levels. B2M mutations and the status of TP53 in baseline samples were obtained from Dr. Brian Walker, as described previously [50].

Two other RNA-seq studies with normal plasma cells were retrieved from the Gene Expression Omnibus (GEO). Data of bone marrow-derived plasma cells from five healthy individuals and five newly diagnosed MM patients were obtained from GSE110486 [51]. Data of plasma cells from bone marrow or tonsil of another eight normal subjects were acquired from GSE114816 [52].

For all RNA-seq data, an initial sequence-level quality assessment was performed using FastQC (v0.11.5). The alignment-free quantification tool Salmon (v1.2.1) [53] was used to quantify the expression of gene transcripts from RNA-seq data using the reference transcriptome built from Gencode (GRCh38, v32) gtf annotation as the index. The gene-level transcript abundance was calculated using the *tximport* package in R.

The normalized gene expression data of 887 cancer cell lines (dated 2018.09.29) and their annotations (dated 2018.12.26) were downloaded from the Cancer Cell Line Encyclopedia (CCLE) [54] data portal (https://portals.broadinstitute.org/ccle/data).

### Identification of intron retention events

To quantify the IR events for MM samples, RNA-seq reads were aligned to the GRCh38 reference genome using STAR (v2.7.2) [55]. Uniquely mapped RNA-seq reads were used to quantify the expression levels of retained introns using HTseq [56] package. Additional criteria were applied to filter the identified IR events: (1) read counts for both the intron region and its flanking exon regions were > 10; (2) read coverage of the intron was comparable to its flanking exons, such that the transcripts per million (TPM) ratio of introns to flanking exons was > 0.05 and < 0.5. MM-specific IR events were further selected by removing the events that were observed in normal plasma cells using the same filtering criteria.

### IR-neoAg prediction

We used *arcasHLA* (v1.1) to infer HLA class-I genotypes from the RNA-seq data [57]. Sequences from the retained introns were translated into peptides by extending the open reading frame of the upstream exon using the standard codon table. The translated peptides were segmented into 8 to 11 amino acid lengths that contained at least one intron-encoded amino acid. For each patient, NetMHCpan4.1 was used to estimate the binding affinity of the IR-derived neopeptides with the patient’s HLA alleles [58]. A binding affinity rank score less than 2 (default parameter of NetMHCpan4.1) was regarded as a neoantigen candidate. Expression levels for each neoantigen were determined by the abundance of IR events that generated the specific neoantigen.

### Differential expression and pathway enrichment analysis

Differentially expressed genes in plasma cells between MM and healthy bone marrow samples were identified using the *limma* [59] package in R. A total of 1790 gene sets from MsigDB version 7.2, including KEGG, REACTOME, and HALLMARK gene sets, were used for enrichment analysis. Fisher’s exact test was used to test for pathway enrichment significance, and the p-value was adjusted for multiple hypothesis correction using the Bonferroni method [60]. *ClusterProfiler* was used to visualize the pathway enrichment results [61]. Single-sample gene set enrichment analysis (ssGSEA) was used to assess the pathway activity in each individual using GSVA [62] package in R, using default parameters.

### Cell culture of MM cells and spliceosome inhibition

KMS11, U266, JJN3, AMO1 MM cell lines were kindly provided by Dr. David Roodman and cultured at 37 °C in a humidified atmosphere containing 5% CO_2_ and maintained in RPMI media supplemented with 10% fetal bovine serum (FBS). Cells were tested for mycoplasma infection monthly as a regular lab routine. Pladienolide-B (Cayman Chemical Company, Ann Arbor, MI; cat# 16538) was dissolved in dimethyl sulfoxide and used at the following concentrations: 0, 0.1, 1, 5, 10, and 100 nM. Flow-cytometric analyses were performed at 96 h post-treatment, two independent biological replicates were analyzed for each treatment.

### Antibodies and flow cytometry analysis

The following flow-cytometry antibodies were used: HLA-DR, DQ, DP-APC (Biolegend, San Diego, CA cat# 361714), Isotype control-APC (Biolegend cat# 400222), MHC class I-PE (LSBio, Seattle, WA cat# LS-C751033-0.1). Isotype control-PE (Abcam, Waltham, MA cat# ab91357). Flow cytometric data were acquired using the LSR II flow cytometer (BD Biosciences, San Jose, CA) and analyzed with FlowJo software.

### Statistical considerations

Survival analysis and Cox-proportional hazard comparison were performed using the R package *Survival* with log-rank test and hazard ratio statistical tests [63]. Significant differences in the value of the two given groups were assessed using the Mann-Whitney-Wilcoxon test [64]. Statistical analyses were performed in R (v4.0.2).

## Supplementary information

Supplementary file

Table S1

Table S2

## Data Availability

The computational algorithm and source code allowing for reproduction of the intron retention-induced neoantigen quantification in this manuscript are available at https://github.com/cpdong/IntronNeoantigen.
