# Supplementary material for: Intron retention-induced neoantigen load correlates with unfavorable prognosis in multiple myeloma
Source: Oncogene. 2021 Sep 9;40(42):6130–8. doi: 10.1038/s41388-021-02005-y (PMC8426332; doi:10.1038/s41388-021-02005-y)
Supplement: Supplementary file 1 — Supplementary file [file 41388_2021_2005_MOESM1_ESM.pdf]

Figure S1

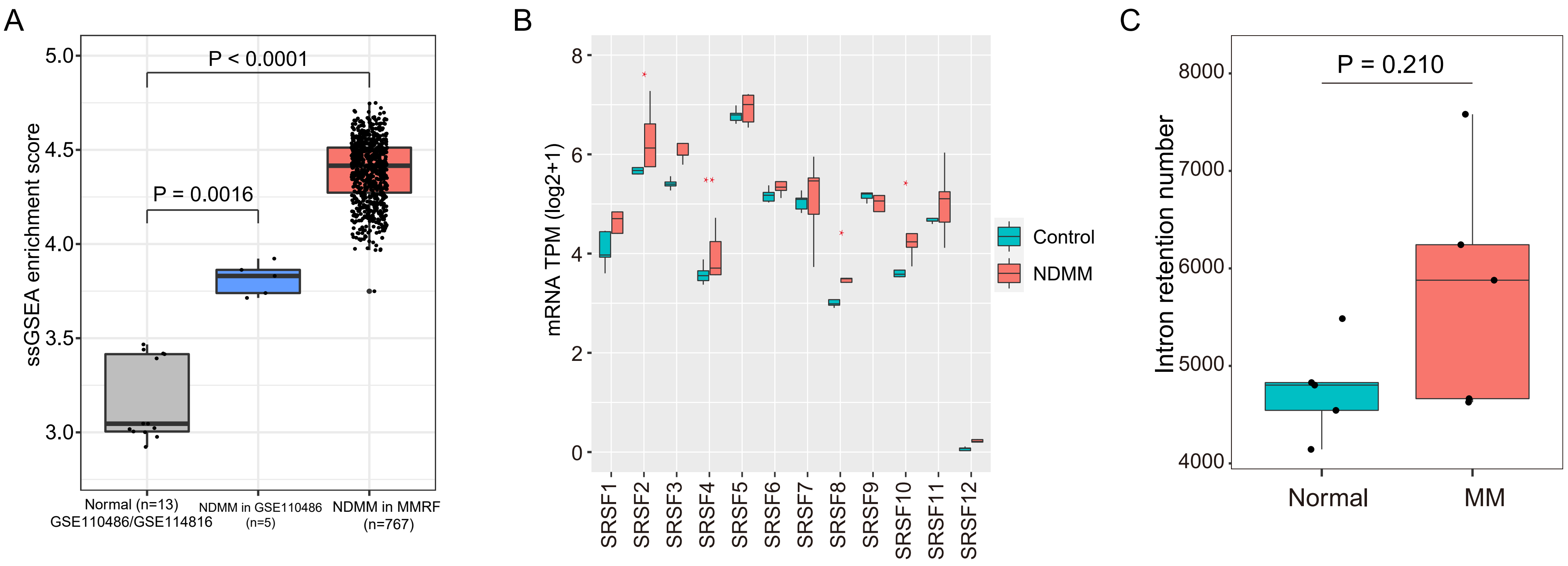

Figure S1. Plasma cells from newly diagnosed MM (NDMM) patients show higher levels of differential gene expression, upregulation of splicing factor genes and more intron-retention (IR) events compared to normal plasma cells. **A** Single-sample gene set enrichment analysis (ssGSEA) of the 230 upregulated genes in the MMRF newly diagnosed MM (NDMM) patient cohort . The enrichment scores for the NDMM samples in the MMRF data were significantly higher than the 13 normal plasma samples (5 in GSE110486 and 8 in GSE114816), and were also higher than the 5 NDMM samples in GSE110486. **B** Comparison of transcripts per million (TPM) of serine and arginine-rich splicing factor (SRSF)protein genes from NDMM (GSE110486) and normal plasma (control) RNAseq data. Asterisks denote significant differences in expression (*, p-value < 0.05 and **, p-value <= 0.01). **C** Comparison of the number of IR events in normal plasma cells and plasma cells from NDMM patients from GSE110486 (n = 5 per group). P values were determined using the Mann-Whitney test. Box plots show the median and 25th and 75th percentiles (box) and the 95% confidence interval (whiskers).

Figure S2

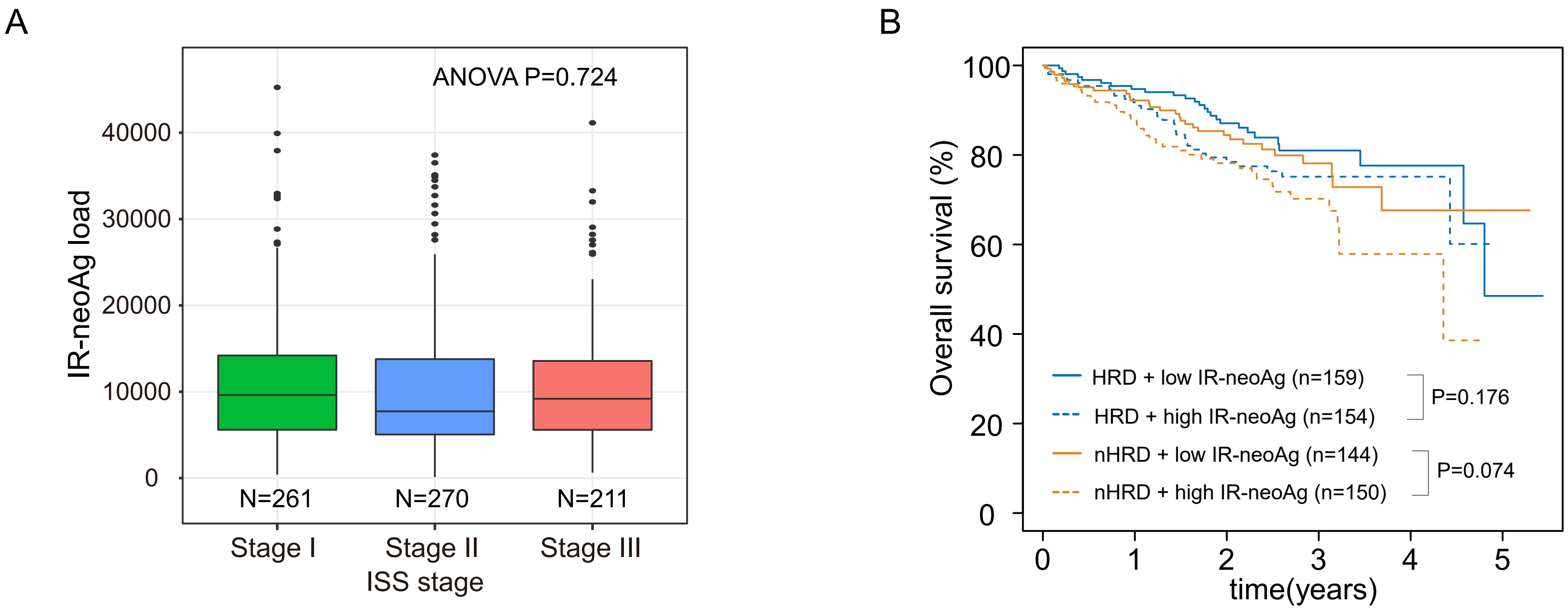

Figure S2. IR-neoantigen load correlated with unfavorable clinical outcome of newly diagnosed MM. **A** Distribution of the IR-neoantigen load in MM patients with different ISS stages. **B** Kaplan–Meier survival curves show overall survival in newly diagnosed MM patients with hyperdiploidy (HRD) or nonhyperdiploid (nHRD) and low or high IR-neoantigen load.

Figure S3

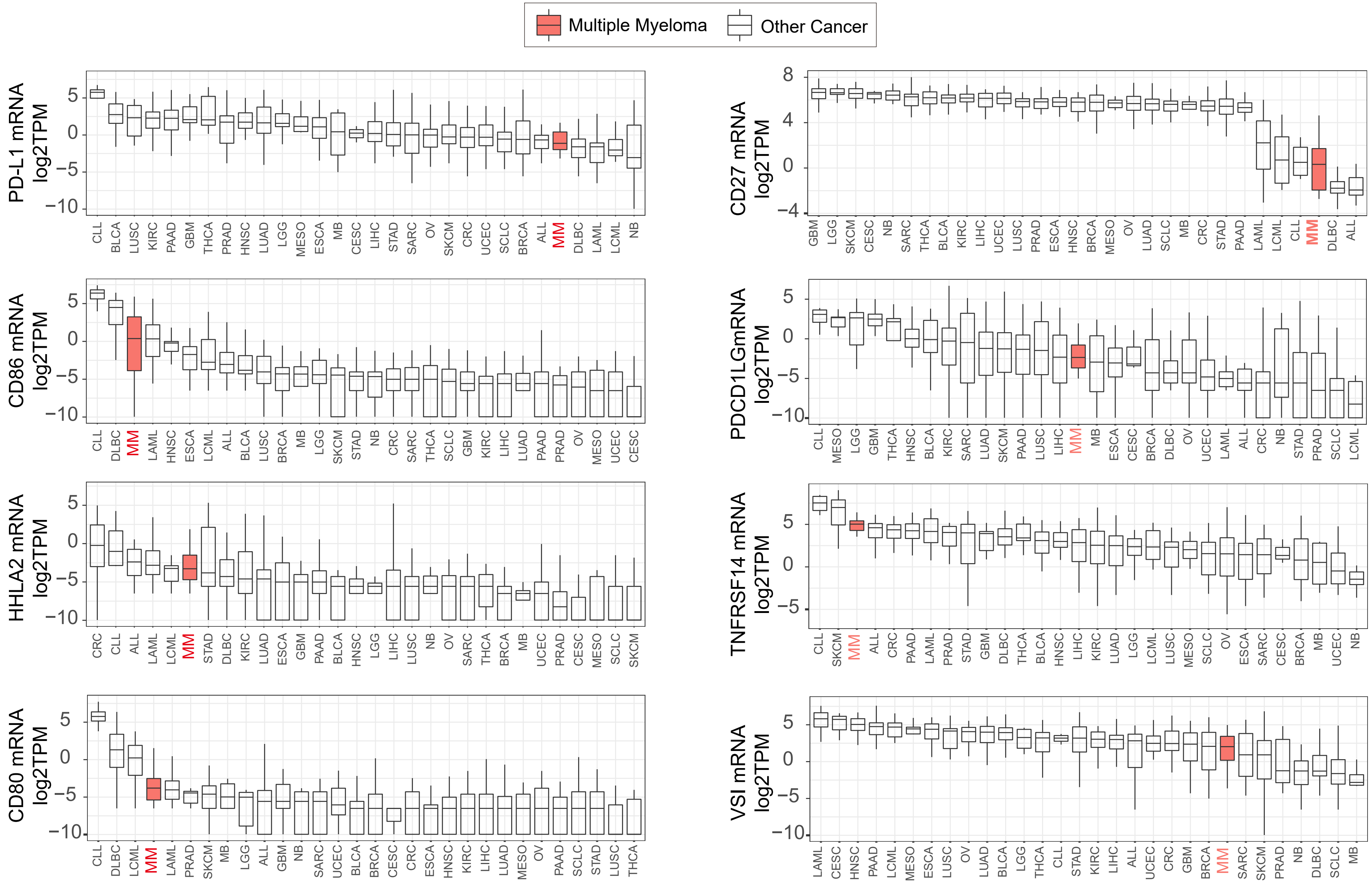

Figure S3. Comparison of RNA expression of theB7 ligand genes in MM vs. other cancer cell lines from the Cancer Cell Line Encyclopedia (CCLE) database. The red box represents the 29 MM cell lines in CCLE.

Figure S4

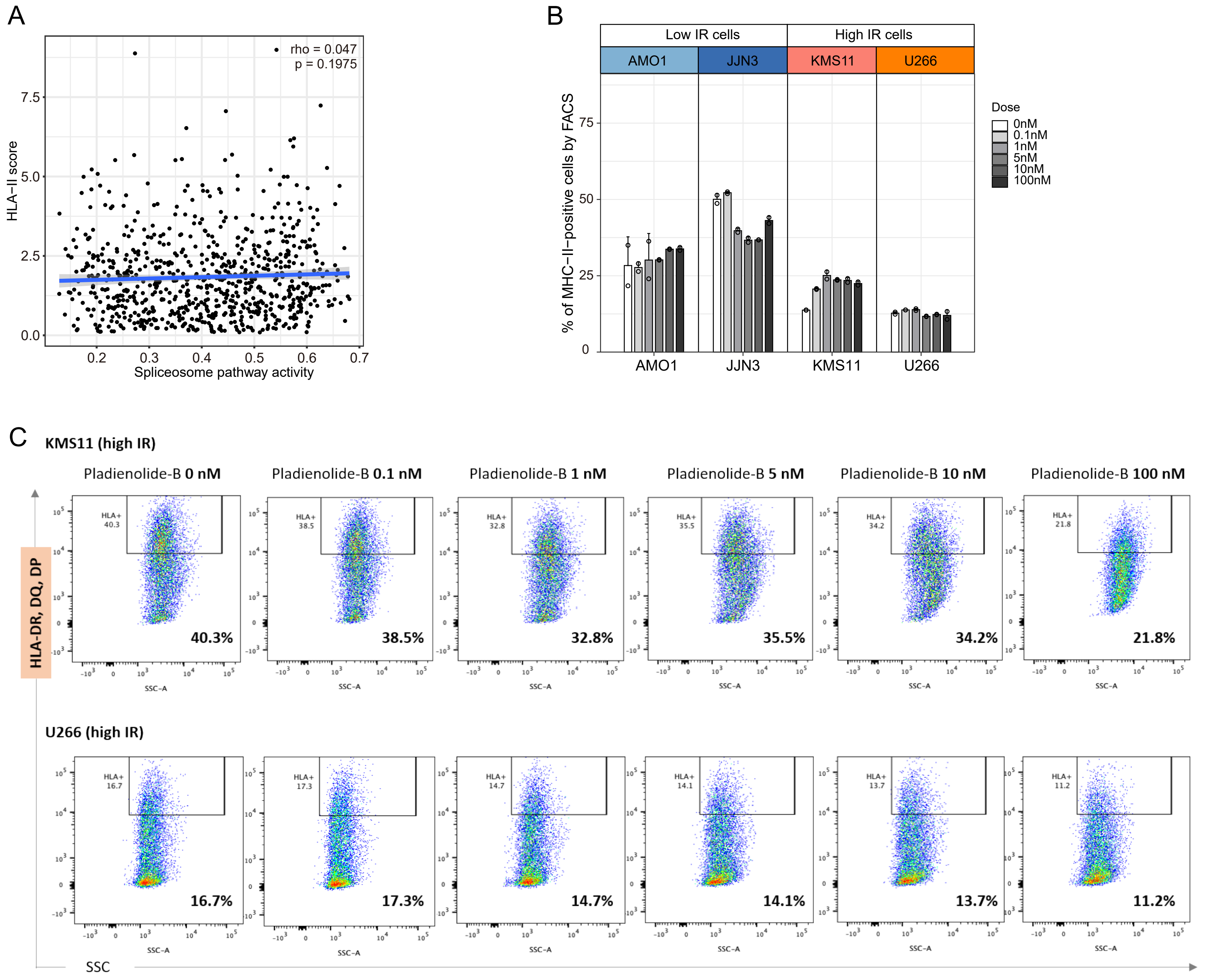
A
rho = 0.047
p = 0.1975
HLA−II score
Spliceosome pathway activity
B
Low IR cells
High IR cells
AMO1
JJN3
KMS11
U266
% of MHC−II−positive cells by FACS
Dose
0nM
0.1nM
1nM
5nM
10nM
100nM
C
KMS11 (high IR)
Pladienolide-B 0 nM
Pladienolide-B 0.1 nM
Pladienolide-B 1 nM
Pladienolide-B 5 nM
Pladienolide-B 10 nM
Pladienolide-B 100 nM
HLA-DR, DQ, DP
40.3%
38.5%
32.8%
35.5%
34.2%
21.8%
U266 (high IR)
16.7%
17.3%
14.7%
14.1%
13.7%
11.2%
SSC-A
SSC

Figure S4. MHC-II expression levels were defined in KMS11, U266, JJN3 and AMO1 MM cell lines by flow cytometry. While significant differences were observed between cells bearing high IR (KMS11 and U266) and cells bearing low IR (JJN3 and AMO1), no significant differences in MHC-II abundance were observed following spliceosome inhibitor treatment for 96 hours. **A** HLA class II gene scores (average expression of HLA-DPA1/DPB1/DQA1/DQB1/DRB1 alleles) from MMRF RNA-seq data were not associated with spliceosome pathway activities. **B** MHC-II cell surface levels were not changed in either high or low IR MM cell lines following pladienolide-B treatment. **C** Representative flow cytometry charts illustrating the percentage of KMS11 or U266 cells with MHC-II (HLA-DR/DP/DQ) gene expression following pladienolide-B treatment. Six pladienolide B concentrations (0, 0.1, 1, 5, 10, 100 nM) were tested and flow-cytometric analyses were performed at 96 hours. Gates were set based on isotype controls and unstained controls.

Table S3. List of 20 T-cell signaling co-inhibitory genes using in this study

Gene	Common_name	Receptor_gene	Immune_checkpoint_function	Family
CD80	B7-1	CD28, CTLA4	Inhibitory(CTLA4)/Stimulatory(CD28)	B7
CD86	B7-2	CD28, CTLA4	Inhibitory(CTLA4)/Stimulatory(CD28)	B7
CD274	PD-L1	PDCD1	Inhibitory	B7
PDCD1LG2	PD-L2	PDCD1	Inhibitory	B7
CD276	B7-H3	unknown	Inhibitory	B7
VSIR	C10orf54/VISTA/PD-1H/B7-H5	unknown	Inhibitory	B7
HHLA2	B7-H7	TMIGD2	Inhibitory	B7
TNFRSF14	HVEM	BTLA, CD160, TNFSF14	Inhibitory	B7
PVR	CD155	CD226, TIGIT, CD96	Inhibitory	CD226
NECTIN2	PVRL2/CD112	CD226, TIGIT	Inhibitory	CD226
NECTIN3	PVRL3/CD113	TIGIT	Inhibitory	CD226
BTN1A1		unknown	Inhibitory	Butyrophilins
BTN2A2		unknown	Inhibitory	Butyrophilins
BTNL2		unknown	Inhibitory	Butyrophilins
IDO1		NA (enzyme)	Inhibitory	Enzyme
IDO2		NA (enzyme)	Inhibitory	Enzyme
TDO2		NA (enzyme)	Inhibitory	Enzyme
NT5E	CD73	NA (enzyme)	Inhibitory	Enzyme
ENTPD1	CD39	NA (enzyme)	Inhibitory	Enzyme
ARG1		NA (enzyme)	Inhibitory	Enzyme
